# Impact of structural biologists and the Protein Data Bank on small-molecule drug discovery and development

**DOI:** 10.1016/j.jbc.2021.100559

**Published:** 2021-03-18

**Authors:** Stephen K. Burley

**Affiliations:** Research Collaboratory for Structural Bioinformatics Protein Data Bank, Institute for Quantitative Biomedicine, Rutgers, The State University of New Jersey, Piscataway, New Jersey, USA; Department of Chemistry and Chemical Biology, Rutgers, The State University of New Jersey, Piscataway, New Jersey, USA; Rutgers Cancer Institute of New Jersey, Robert Wood Johnson Medical School, New Brunswick, New Jersey, USA; Research Collaboratory for Structural Bioinformatics Protein Data Bank, San Diego Supercomputer Center, University of California, San Diego, La Jolla, California, USA; Skaggs School of Pharmacy and Pharmaceutical Sciences, University of California, San Diego, La Jolla, California, USA

**Keywords:** structural biology, protein structure, open-access biodata resource, FAIR principles, Protein Data Bank, PDB50, RCB Protein Data Bank, drug discovery and development, drug target validation, structure-guided drug discovery, 3D, Three-dimensional/Three dimensions, 3DEM, Three-dimensional Electron Microscopy, Abl, Abelson Proto-oncogene 1, Nonreceptor Tyrosine Kinase, ADME, Absorption-Distribution-Metabolism-Excretion, BMRB, Biological Magnetic Resonance Data Bank, CDK4, Cyclin-dependent Kinase 4, CDK6, Cyclin-dependent Kinase 6, CML, Chronic Myelogenous Leukemia, D3R, Drug Design Data Resource, DNA, Deoxyribonucleic Acid, EMDB, Electron Microscopy Data Bank, FAIR, Findability, Accessibility, Interoperability, and Reusability, FDA, Food and Drug Administration, GIST, Gastrointestinal Stromal Tumor, HIV, Human Immunodeficiency Virus, ID, Identifier, IDH2, Isocitrate Dehydrogenase 2, mmCIF, Macromolecular Crystallographic Information File, MX, Macromolecular Crystallography, NMR, Nuclear Magnetic Resonance, OneDep, Unified wwPDB System for Deposition, Biocuration, and Validation, PDB, Protein Data Bank, PDBe, Protein Data Bank in Europe, PDBj, Protein Data Bank Japan, PDBx, Protein Data Bank Exchange Data Dictionary, PH+, Philadelphia Chromosome Positive, QT Interval, Electrocardiogram Q-wave to T-wave Interval, RCSB, Research Collaboratory for Structural Bioinformatics, RNA, Ribonucleic Acid, Src, Proto-oncogene Sarcoma Tyrosine-protein Kinase, US, United States, wwPDB, Worldwide Protein Data Bank

## Abstract

The Protein Data Bank (PDB) is an international core data resource central to fundamental biology, biomedicine, bioenergy, and biotechnology/bioengineering. Now celebrating its 50th anniversary, the PDB houses >175,000 experimentally determined atomic structures of proteins, nucleic acids, and their complexes with one another and small molecules and drugs. The importance of three-dimensional (3D) biostructure information for research and education obtains from the intimate link between molecular form and function evident throughout biology. Among the most prolific consumers of PDB data are biomedical researchers, who rely on the open access resource as the authoritative source of well-validated, expertly curated biostructures. This review recounts how the PDB grew from just seven protein structures to contain more than 49,000 structures of human proteins that have proven critical for understanding their roles in human health and disease. It then describes how these structures are used in academe and industry to validate drug targets, assess target druggability, characterize how tool compounds and other small-molecules bind to drug targets, guide medicinal chemistry optimization of binding affinity and selectivity, and overcome challenges during preclinical drug development. Three case studies drawn from oncology exemplify how structural biologists and open access to PDB structures impacted recent regulatory approvals of antineoplastic drugs.

This invited contribution to the Protein Data Bank (PDB)’s 50th anniversary celebration issue of the *Journal of Biological Chemistry* summarizes my perspective on the impact of structural biologists and open access to data from the PDB (1, 2) on discovery and early-stage development of small-molecule therapeutic agents. The PDB is universally recognized as being comprehensive, authoritative, **FAIR** (Findable, Accessible, Interoperable, and Reusable (3)), and trustworthy (https://coretrustseal.org). It is central to research and education in fundamental biology, biomedicine, biotechnology/bioengineering, and energy sciences worldwide (Fig. 1; (4)).

**Figure 1:**
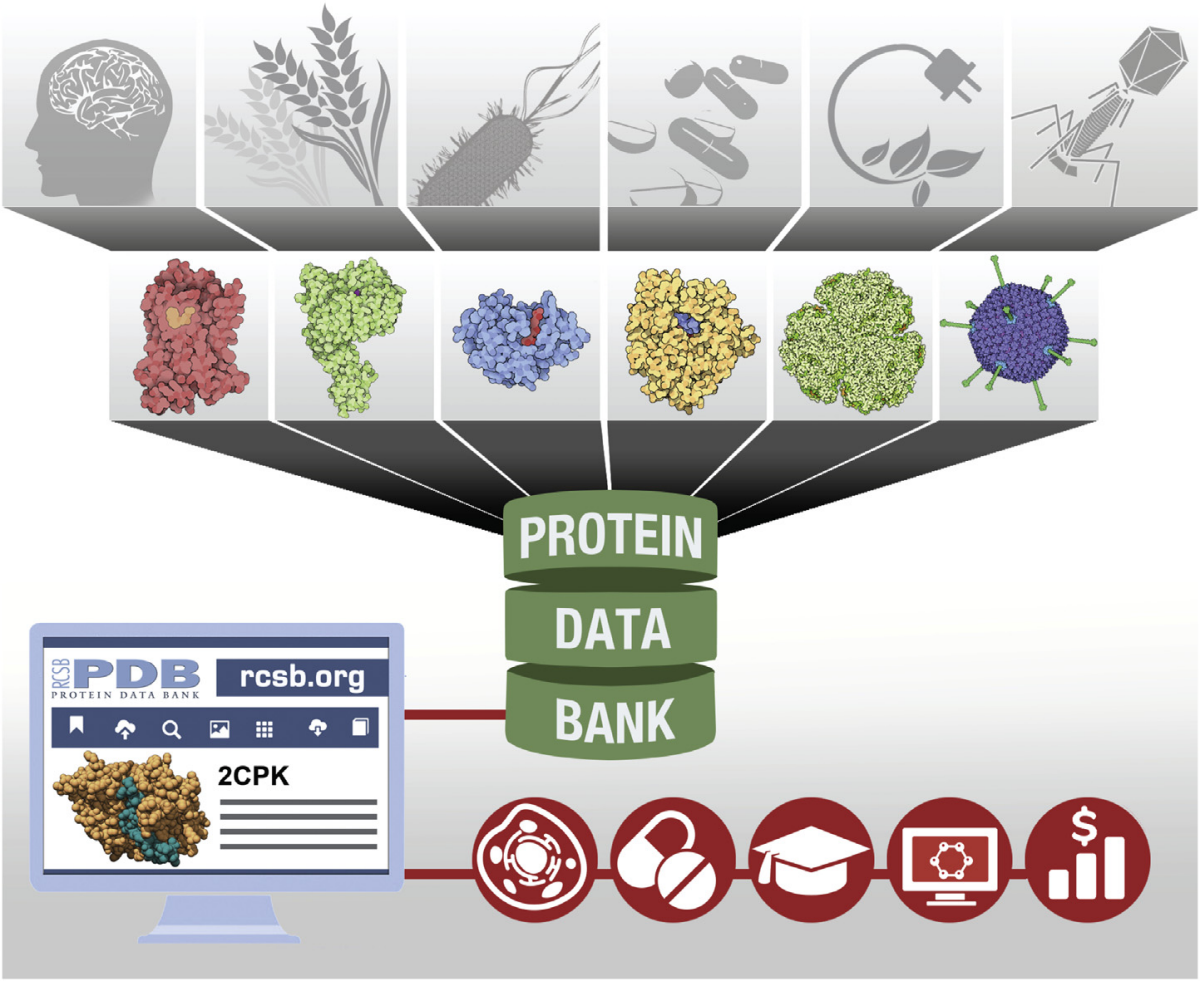
**Availability of the Protein Data Bank archive helps transform 3D structure data to knowledge, benefiting basic and applied research and education**.

Materials for this review were drawn from PDB archive holdings; peer-reviewed publications; anecdotal accounts presented publicly at scientific meetings; my professional experience in academe (Furlaud Professor, The Rockefeller University; Full Investigator, Howard Hughes Medical Institute; University Professor and Henry Rutgers Chair, Rutgers, The State University of New Jersey) and in industry (Chief Scientific Officer, SGX Pharmaceuticals, Incorporated; Distinguished Lilly Scholar, Eli Lilly and Company); and my ongoing roles as Director of the US-funded Research Collaboratory for Structural Bioinformatics Protein Data Bank and coleader of the Worldwide Protein Data Bank (wwPDB) partnership (see below).

I first describe how PDB coverage of human proteins broadened over the past 50 years to encompass more than 49,000 structures of human proteins and then describe PDB infrastructure that directly supports structural biologists and drug hunters (and indirectly all PDB data consumers). I go on to explain how PDB structures are being used in academe and industry to validate drug targets, assess target druggability, characterize how tool compounds and other small molecules bind to drug targets, guide medicinal chemistry optimization of binding affinity and selectivity, and overcome challenges during preclinical drug development. Finally, some quantitative information on the impact of PDB structures on drug discovery and development is presented together with illustrative case studies focused on recently approved antineoplastic agents.

For the avoidance of doubt, I freely acknowledge that structural sciences and 3D structure information are but two of the many bioscience tools/data that underpin drug discovery and early-stage drug development. Other essential elements of the drug hunting ecosystem include biochemistry, biophysical measurements, genome sequencing, molecular and cellular biology, bioimaging and mass spectrometry instrumentation, high-throughput biochemical and cell-based screening, medicinal chemistry, computational chemistry, molecular dynamics simulations, pharmacology, toxicology, statistics and machine learning, *etc*.

## Growth of the PDB archive

As of December 31, 2020, PDB holdings had grown nearly 25,000-fold to 173,000 structures from just seven macromolecular crystallography (MX) structures in 1971. The PDB is currently growing at the rate of ∼10% per year. During 2020, 14,058 new structures were released by the PDB archive. The vast majority of these structures continue to come from MX (∼80%), with 3D electron microscopy (3DEM; (5)) adding ∼17%, and nuclear magnetic resonance (NMR) spectroscopy contributing 3%.

Growth of the PDB was far from linear over its first 50 years. The decade between 1991 and 2000 saw explosive growth (∼27-fold) in the PDB archive from 507 structures (as of December 31, 1990) to 13,589 by the end of 2000. This period coincided with synergistic convergence of five critical technological advances underpinning MX, including synchrotron beamlines (6), routine diffraction measurements at cryogenic temperatures (7), X-ray reflection phasing using anomalous scattering (8), facile *E. coli* expression of exogenous proteins (9), and simulated annealing structure refinement against measured structure factors (10). This period also coincided with a sea change in data sharing practices within the global structural biology community; one that was a decade or more ahead of other scientific disciplines. A grassroots movement led by a cadre of prominent structural biologists influenced scientific journals and research funders to require PDB deposition of atomic coordinates as condition for publication or funding of structural biology research. Later in 2008, PDB deposition of MX experimental data became mandatory, which was essential for making validation of 3D structures more stringent (see below). Subsequently, deposition of experimental data for structures determined using NMR spectroscopy and 3DEM became mandatory.

## Human protein structure coverage in the PDB archive

Among drug hunters, there is an understandably strong preference for structures of the actual drug target proteins, be they human, bacterial, or viral. Even small differences in amino acid sequence (hence 3D structure and biochemical function) can be misleading during medicinal chemistry optimization of small-molecule drug candidates. The inaugural human protein structure deposited to the PDB in 1976 was that of a Bence-Jones immunoglobulin light-chain dimer (PDB ID: 1rei (11)). By the end of 1980, PDB holdings included only five human proteins (∼7% of archival contents), four of which were unique. One of these structurally characterized proteins, prealbumin (a.k.a. transthyretin, PDB ID: 2pab (12)), was subsequently validated as the target of tafamidis, a small-molecule drug (molecular weight or MW < 1000 Da) approved by the United States (US) Food and Drug Administration (FDA) in 2019 for treatment of adults diagnosed with cardiomyopathy due to transthyretin-mediated amyloidosis. Over the next decade, only 34 human protein structures were added to the archive. Four of them were subsequently validated as targets of approved small-molecule drugs, including hemoglobin S (PDB ID: 1hbs (13)), carbonic anhydrase 2 (PDB ID: 2ca2 (14)), elastase (PDB ID: 1hne (15)), and dihydrofolate reductase (PDB ID: 1dhf (16)).

Coincident with explosive growth in the PDB from 1991 to 2000, archival holdings of human protein structures grew nearly 68-fold to 2644. Advances in exogenous protein overexpression in insect and mammalian cell hosts further accelerated the pace of structure determination for human proteins in the 2000s (17). By the end of 2010, the archive included 16,640 human protein structures. Among the most exciting of these new structures were the first examples of human G-protein-coupled receptors (reviewed in (18)), which represent an important class of drug targets (19, 20).

As of December 31, 2020, PDB holdings of human protein structures had grown to 49,723 (∼29% of the archive). In 2020, additions of first examples of human protein structures numbered 1430. This annual growth metric, reflecting ongoing increases in structural coverage of the human proteome, consistently exceeded 1000 structures/year from 2016 through 2020. Given the reductionist approach taken by many structural biologists, particularly those using either MX or NMR, these metrics should not be conflated with per residue coverage of the human proteome. SwissModel (21) provides useful tools for assessing structural coverage of human proteins and the impact of homology modeling on our knowledge of the human proteome in 3D (see https://swissmodel.expasy.org/repository/species/9606).

## PDB infrastructure supporting structural biologists and drug discovery

Since 2003, the PDB has been managed jointly by the wwPDB partnership (https://wwPDB.org; (2, 22)), including the US-funded Research Collaboratory for Structural Bioinformatics Protein Data Bank or RCSB PDB (https://RCSB.org; (19, 23, 24)), Protein Data Bank in Europe or PDBe (https://PDBe.org; (25)), Protein Data Bank Japan or PDBj (https://PDBj.org; (26)), and Biological Magnetic Resonance Data Bank or BMRB (https://BMRB.io; (27)).

The wwPDB OneDep software system (https://deposit.wwpdb.org) for deposition, validation, biocuration of incoming structures supports PDB data depositors worldwide. OneDep helps to ensure that every structure coming into the archive is rigorously validated and expertly biocurated (28, 29, 30). The OneDep system is also used for periodic remediation of PDB data (31, 32, 33, 34). Joint wwPDB management of the archive depends critically on active and productive engagement with community thought leaders on matters pertaining to data standards and validation of both structures and supporting experimental data and metadata. wwPDB Validation Task Forces (35, 36, 37) and specialized workshops (38) have played important roles in developing community-endorsed validation tools that help ensure archive data quality, accuracy, and integrity. Every structure in the PDB archive is accompanied by an official, public wwPDB validation report that drug hunters and other PDB data consumers can rely on to assess the reliability of the atomic coordinates of the macromolecule and any bound ligands.

Data stored in the PDB archive conform to the PDBx/mmCIF data dictionary (39, 40). In 2014, PDBx/mmCIF became the internationally recognized, official data and metadata standard for the PDB (41). The RCSB PDB (with wwPDB partners and the wwPDB PDBx/mmCIF Working Group) coordinates PDBx/mmCIF development and hosts a public repository for data standards, metadata specifications, tutorials, and links for accessing relevant software tools (http://mmcif.wwpdb.org). The PDBx/mmCIF framework enables automated checking of data consistency. PDB chemical and molecular data (33, 42) are also managed with PDBx/mmCIF (https://www.wwpdb.org/data/ccd; https://www.wwpdb.org/data/bird). Use of this fully extensible, machine-readable data standard helps to ensure that PDB data are **FAIR**. Provenance and quality information for each data item are faithfully preserved throughout all wwPDB deposition, biocuration, validation, and remediation procedures. Since 2017, the PDB archive has supported versioning, allowing replacement of atomic coordinates by the Depositor of Record when errors, *etc*., require correction (43).

Within the PDBx/mmCIF schema, the wwPDB Chemical Component Dictionary (CCD) (42) is of particular importance to drug hunters. Every ligand (*i.e.*, Chemical Component) represented in the PDB archive (including amino acids, nucleotides, organic compounds, and ions) is defined in the CCD. It currently houses >32,500 unique ligands. The CCD is used to standardize atom nomenclature and chemical naming using 2D graphic matching during OneDep biocuration (30). In addition, ligand geometry and chirality checking is performed using Mogul (44) (courtesy of the Cambridge Crystallographic Data Centre (CCDC) (45), as recommended by wwPDB X-ray VTF (35)). wwPDB validation software is periodically updated to incorporate recommendations from community experts. Most recently, new ligand validation tools of particular benefit to drug hunters were added by the wwPDB. They were introduced in response to recommendations from the wwPDB/CCDC/D3R Ligand Validation Workshop (38) aimed at improving the validation and ultimately the quality of cocrystal structures in the PDB (46).

## Utility of PDB structures for small-molecule drug discovery and development

Over the past two decades, structural biologists and structure-guided drug discovery have become firmly established within the biopharmaceutical industry (47, 48). 3D structures can explain how small-molecule ligands bind to their target proteins (*e.g.*, imatinib targeting Abl in chronic myeloid leukemia (PDB ID: 1iep; (49, 50))). Structural data have also proven useful in overcoming some of the myriad challenges inherent in turning biochemically active compounds into potent drug-like molecules suitable for safety and efficacy testing in animals and humans (51). Most, probably all, major biopharmaceutical companies around the world maintain a copy of the PDB archive within their firewall. Bringing PDB data inside the firewall enables interoperation of public-domain PDB structures with proprietary structures generated at each of the companies. Conservative estimates suggest that proprietary protein structures held as trade secrets inside company firewalls are comparable in aggregate number to the current size of the PDB. Willingness on the part of industry structural biologists (and company leadership) to contribute some of these data to the PDB is both much appreciated and essential for fostering ongoing technical innovations within the experimental and computational ecosystems that make structure-guided drug discovery possible (and successful).

Public-domain 3D structure data archived in the PDB are used in small-molecule drug discovery and early-stage drug development at five points within the process.

### Target biology

Function follows form in biology. Atomic-level 3D structures made freely available from the PDB provide functional insights that are not always readily apparent from amino acid sequence (reviewed in (4, 20)). Simply put, there is no substitute for a direct look at the 3D structure of a potential drug discovery target. This information helps researchers understand how it works at the atomic level (*i.e.*, molecular mechanism) and how it contributes to human health and disease. Equally important, use of 3D structures to interpret the results of human genome sequencing studies (*e.g.*, driver mutations specific to tumors, genome-wide associations) influences target selection in many therapeutic areas. Industry colleagues have frequently remarked to me that “the first thing they do when starting a new drug-discovery project is to search the PDB and look hard at potential target structure(s).”

Developing the fullest possible understanding of the role that a given drug discovery target plays in human health and disease represents a critical determinant of success. An influential industry-wide analysis of the causes of attrition during drug discovery and development campaigns documented that efficacy failures account for an overall attrition rate of nearly 30% (52). A more recent analysis identified lack of efficacy as the cause of up to 66% of failures during Phase II clinical trials (53). Efficacy failures occur when the drug candidate engages the target, achieves the desired biochemical end point (*e.g.*, enzyme inhibition) without serious adverse events, yet fails to deliver the desired clinical benefit. Hence, the importance of gaining comprehensive knowledge of target biology well before embarking on expensive and lengthy human clinical trials.

### Target druggability

3D structures enable visualization of surface features (*e.g.*, clefts, invaginations) likely to bind small organic compounds and thereby inhibit enzyme action or some other biochemical or biological function (reviewed in (54)). Much of the free energy of binding of small molecules to proteins comes not from the enthalpic contribution (ΔH), but from the entropic contribution (-TΔS) to the Gibbs free energy change (ΔG=ΔH-TΔS) of the system upon ligand binding. Higher-resolution MX and 3DEM structures of proteins frequently reveal low-entropy water molecules occupying concave surface features. When small-molecule ligands bind within such clefts, they displace most, if not all, of the bound water molecules expelling them into the bulk solvent and increasing the entropy of the system. It is, therefore, widely believed to be more difficult to discover small molecules that target flatter, relatively featureless protein surfaces (*e.g.*, protein–protein interaction interfaces) *versus* deeply invaginated clefts characteristic of enzyme active sites (55). Prequalifying a protein as a target amenable to a small-molecule drug(s) using 3D structure information can increase the probability of finding suitable lead compounds and thereafter reduce the likelihood of attrition during medicinal chemistry optimization.

### Small-molecule binding

Public-domain cocrystal structures frequently provide useful precompetitive information concerning binding of tool compounds to potential drug discovery targets (56). Even more powerful are the many cocrystal structure studies carried out within biopharmaceutical companies that directly assess in 3D how small-molecule hits coming from biochemical or cell-based assays (57) or biophysical measurements (58) bind to would-be drug targets. *In silico* virtual screening exercises carried out computationally with millions of small molecules can also provide useful information regarding potential starting points for medicinal chemistry.

When it comes to understanding the chemotype binding properties of a would-be drug target, the preferred method is fragment screening (59). This approach was pioneered in the 2000s by a number structure-guided drug discovery companies, including Astex Pharmaceuticals, Plexxikon, and SGX Pharmaceuticals, Inc. It typically involves soaking of preformed protein crystals with one or more low-molecular-weight chemical fragments (MW∼150–300; frequently referred to as scaffolds) and then using high-throughput X-ray crystallography to visualize any bound fragments. Hit rates for fragment screening campaigns are typically 1 to 5%, permitting efficiency gains by using compound mixtures (60). Following fragment screening, a subset of the bound scaffolds occupying the desired site on the surface of the target (*e.g.*, enzyme active site) are selected for chemical elaboration on the bases of patent novelty, synthetic tractability, *etc*. Today, fragment screening is widely used throughout the biopharmaceutical industry (59). In some organizations, fragment libraries are prescreened against target proteins using biophysical tools such as solution NMR spectroscopy (61) and surface plasmon resonance (62). Successful prosecution of fragment/scaffold hits detected by whatever means, however, almost invariably depends on repeated access to cocrystal structures of bound compounds during medicinal chemistry lead optimization.

Typical fragment or scaffold libraries consist of 500 to 5000 compounds, each with multiple reactive sites capable of supporting automated or semiautomated chemistry with large numbers (*i.e.*, sometimes tens of thousands) of commercially available modifying substituents. Well-designed fragment libraries have the potential to be elaborated into 10^15^ or more unique chemical structure variations (assuming 1000 fragments each with three sites of chemical diversity with 10,000 possible substituents at each reactive site). Thus, the potential chemical diversity of fragment-based approaches to drug discovery far outstrips even the largest compound screening libraries assembled in either academe or industry (typically no more than a few million compounds). Notwithstanding this impressive diversity metric, even the best designed fragment library will never provide access to the enormous number of compounds possible. For reference, the number of distinct molecular structures of MW <500 Da containing only carbon, oxygen, nitrogen, hydrogen, and fluorine atoms that obey the valence rules of chemistry is estimated to be ∼10^60^ (63).

Finally, structural characterization of compound hits detected by any screening method provides valuable insights into how various chemotypes bind (or are predicted to bind) to target proteins. 3D structural information regarding screening hits that are not selected as fragments/scaffolds for medicinal chemistry optimization is frequently used to support decision-making by medicinal chemists (64). Knowledge of the chemotype binding properties of the target site can motivate selection of chemical substituents with which to modify fragment/scaffold hits. This information can also be used later in the optimization process to further optimize the fit of the elaborated lead compound to its binding site.

### Structure-guided lead optimization

PDB structures and ancillary data stored in the archive regarding sample production, crystallization, *etc*., constitute important precompetitive information routinely used by drug hunters. Open access to these data (without limitations on usage) facilitates early-stage drug discovery writ large. Whenever practicable, nearly every the major biopharmaceutical company makes intensive use of cocrystal structures to guide optimization of small-molecule ligand potency from screening hits to lead compounds to drug candidates (reviewed in (47)). In the most favorable cases, knowledge of cocrystal structures of potential off-target proteins (*e.g.*, GSK-3β: inhibition of this protein kinase causes hyperglycemia) can be utilized to help ensure the desired selectivity profile and reduce the likelihood of off-target toxicity. In the absence of experimental cocrystal structures of the target protein, *in silico* docking tools are commonly used to guide lead optimization (reviewed in (65)). Where an experimental 3D structure of the target protein is not available, homology models are routinely combined with these same *in silico* docking tools. Machine learning approaches are also being used with increasing frequency to drive medicinal chemistry campaigns (reviewed in (66)).

Structural guidance of medicinal chemistry decision-making is particularly important when optimizing the physicochemical properties of would-be drug candidates. Lipinski’s “Rule of 5” (67) has often been touted as a basis for determining whether or not a small molecule is “drug like.” Close reading of Lipinski’s landmark paper, however, reveals that the Rule of 5 pertains to oral bioavailability, not drug likeness *per se*. Clinical trial experiences have repeatedly shown that more stringent limits on molecular weight (MW < 400 Da instead of 500 Da) are correlated with increased likelihood of successful outcomes (68). Lower drug candidate lipophilicity (as judged by cLogP, the calculated log_10_ of the partition coefficient between octanol and water) is also correlated with improved clinical trial outcomes (69). Lipophilicity appears to be a critical determinant of nonspecific binding to proteins unrelated to the drug target and consequently unwanted side effects and clinical adverse events. Precise knowledge of how lead compounds bind to target proteins informs decision-making regarding the chemical modifications necessary to maintain cLogP <3 (not <5 as specified in the Rule of 5), while avoiding addition of atoms that increase MW beyond ∼400 Da. Other molecular design considerations influenced by knowledge of 3D structure focus on avoiding synthesis of overly flat compounds, because small molecules lacking Sp3 carbons and chiral centers tend can be poorly soluble in aqueous solution (70).

### Optimization of ADME properties

3D structures of proteins are used to overcome **ADME** (**A**bsorption–**D**istribution–**M**etabolism–**E**xcretion) challenges (reviewed in (51)), typically identified during early-stage (*i.e.*, preclinical) development. More than 500 PDB structures of cytochrome P450 enzymes (Cyp; earliest PDB ID released 2003: 1og2 (71)) help inform additional medicinal chemistry efforts aimed at eliminating Cyp inhibition by drug candidates (or Cyp-mediated metabolism of same) while preserving potent and selective target binding. More than 30 PDB structures of P-glycoprotein multidrug transporters (earliest PDB ID released 2009: 3g5u (72)) are available from the PDB to help medicinal chemists understand and overcome drug resistance due to cellular efflux pumps, which can interfere with the efficacy of cancer chemotherapeutic agents. Finally, three PDB structures of the human ether-a-go-go (commonly referred to as hERG) related potassium channel (earliest PDB ID released 2017: 5va1 (73)) can provide structural insights into potentially life-threatening small-molecule binding that can induce long QT syndrome, bringing with it the risk of sudden death due to the cardiac arrhythmia known as *Torsades des Pointes*.

## Quantitative measures of the impact of structural biologists and the PDB on small-molecule drug discovery

Published case studies (*e.g.*, (74)) and anecdotal accounts presented at scientific meetings leave no doubt as to the importance of contributions to drug discovery made by structural biologists working in the biopharmaceutical industry. Their use of the PDB is corroborated by explicit references to the archive detected in more than 50,000 issued patents and patent applications in process worldwide during 2016 (4).

Given the highly competitive nature of any industry and appropriate concerns of trade secrecy, publication and mandatory PDB deposition of proprietary structures by biopharmaceutical companies often lag the actual research by years. Much of the time, unfortunately, publication never happens because of resource limitations or organizational impediments to making data public. My own biopharmaceutical industry publications describing proprietary research projects attest to the fact that what you see in print and in the PDB is just the tip of the iceberg in terms of the accomplishments of industry structural biologists and their impact on drug discovery (75, 76, 77, 78).

Two quantitative analyses of the impact of structural biologists and PDB structures on drug approvals have been published recently. The first such analysis (79) reported examinations of PDB archival holdings to identify 3D structures relevant to the 171 new small-molecule drugs approved by the US FDA from the beginning of 2010 to the end of 2016. Approximately 92% of these small-molecule drugs (158/171) have known protein targets. In 2018, the PDB archive held 5364 drug/target-related structures containing a known protein target of one of these 171 small-molecule drugs and/or the small-molecule drug itself. In approximately 20% of cases (30/171), the number of PDB structures of the drug target exceeded 100, reflecting the importance of structure-guided approaches for prosecution of certain medicinal chemistry campaigns (*e.g.*, thrombin, earliest PDB ID released 1992: 1fph (80); HIV-protease inhibitors, earliest PDB ID released 1990: 4hvp (81)). This quantitative study also identified 277 PDB structures containing one of the US FDA-approved small-molecule drugs approved between 2010 and 2016.

Freely available structures downloaded from the PDB undoubtedly facilitated a number of these successful drug discovery and development efforts. In most cases, the earliest example of a related PDB structure of the target protein for a given small-molecule drug came from an academic research group. The remainder came from industry or, rarely, from combined academic–industry research teams. More than half of the 5364 drug/target-related structures were deposited to the PDB well before the new drug was approved by the US FDA for clinical use. The median time between earliest deposition of a related PDB structure and US FDA drug approval exceeded 10 years. This metric is significant because development of a newly discovered drug candidate can extend over many years, with the bulk of this time devoted to clinical trials. On average, a new medicine will be 10 to 15 years in the making before regulatory approval (82).

### Bosutinib case study

**Figure 2:**
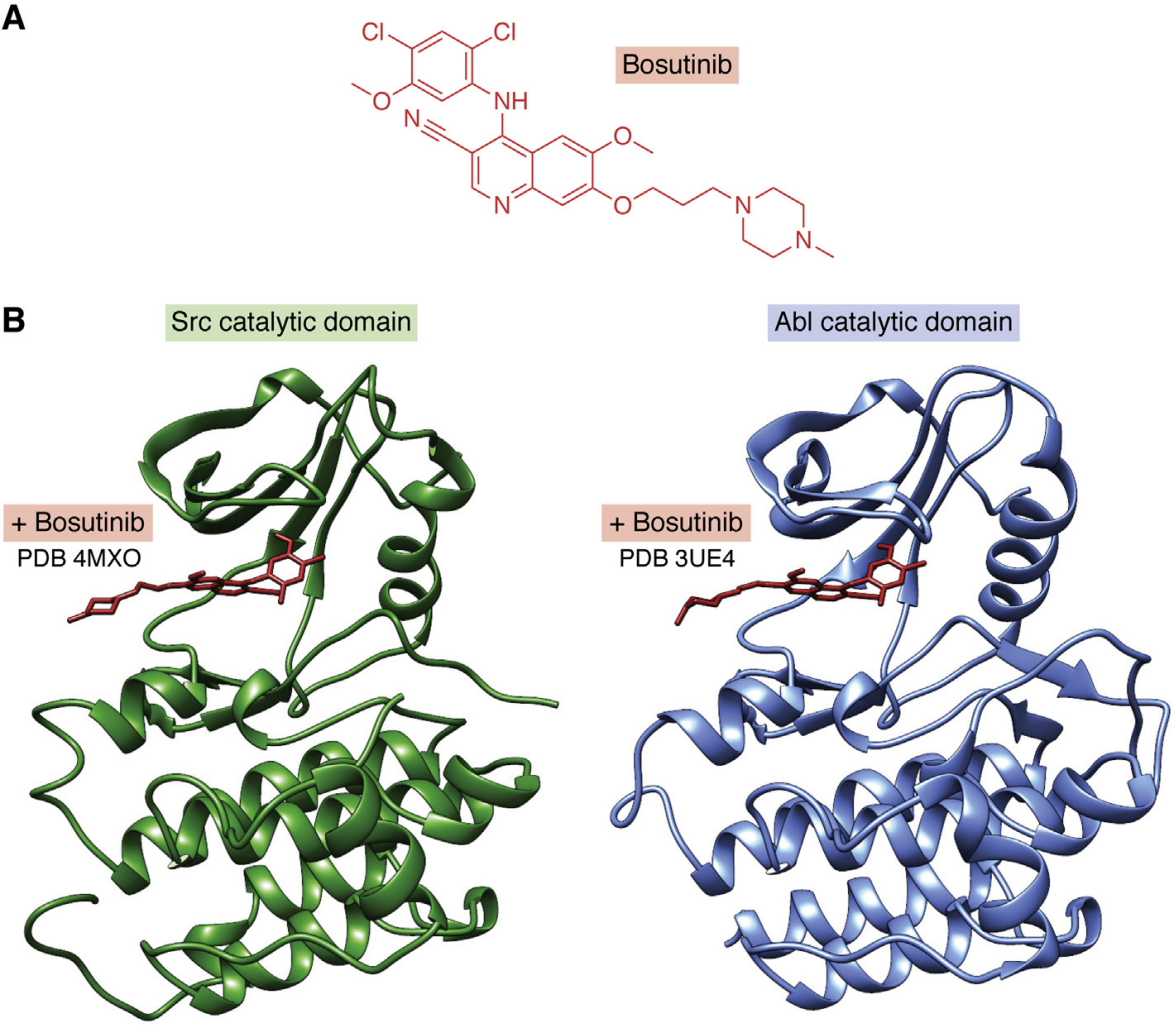
**Bosutinib binding to Src and Abl.***A*, chemical structure of bosutinib. *B*, cocrystal structures of bosutinib (*red*) bound to the catalytic domains of Src (*Left*, *green*; PDB ID: 4mxo (106) and Abl) (*Right*, *blue*; PDB ID: 3ue4 (107)) non-receptor tyrosine kinases.

Bosutinib, a potent inhibitor of the human nonreceptor tyrosine kinases Src and Abl (Fig. 2, *A*), was approved by US FDA in 2012 for treatment of adults with newly diagnosed chronic phase Philadelphia chromosome positive (Ph+) chronic myelogenous leukemia (CML) and chronic, accelerated, or blast phase Ph+ CML with resistance or intolerance to prior therapy. In 2018, the PDB archive held a total of 179 protein structures related to Src or Abl (*e.g.*, wild-type form of bosutinib’s original target Src, mutant forms of Src, phosphorylated forms of Src, bosutinib bound to Src, bosutinib bound to Abl, other small-molecule inhibitors bound to Src, and bosutinib bound to protein kinases other than Src and Abl). Beginning in 1994 with publication and PDB deposition of the earliest structure of human Src (Src homology domain 3 or SH3) by structural biologists at the Glaxo Research Institute (PDB ID: 1shd (83)), PDB structures have provided detailed insights into the biology of Src as a nonreceptor tyrosine kinase, its role as a driver of tumor growth, and inhibition by various small molecules. This work culminated in successful clinical trials of bosutinib targeting Abl in Ph+ CML and regulatory approval. (N.B.: The first PDB structure of the Abl protein kinase catalytic domain determined by an academic research team was released in 2000 (84)). Figure 2, *B* and *C* illustrate the cocrystal structures of bosutinib bound to the catalytic domains of Src and Abl, respectively.

The second quantitative analysis of the impact of the PDB on drug approvals (85) reported examinations of archival holdings to identify 3D structures relevant to the 54 new antineoplastic small-molecule drugs approved by the US FDA from the beginning of 2010 to the end of 2018. The protein targets of every one of these small molecule drugs are known. In 2019, the PDB archive contained 2115 drug/target-related structures containing a known protein target of one of these small molecule drugs and/or the small-molecule drug itself. More than three-quarters of the recently approved small-molecule drugs (41/54, ∼76%) can be found in PDB structures that reveal in 3D at atomic detail how the drug binds to its target protein and, in some cases, to off targets. Virtually all of the 2115 related PDB structures were deposited well before corresponding drug approvals by the US FDA (median time between earliest PDB deposition and regulatory approval exceeded 14 years).

## Evidence for the impact of structure-guided approaches on small-molecule antineoplastic drug approvals

The second quantitative analysis also examined the impact of structure-guided discovery on US FDA approvals of antineoplastic drugs between 2010 and 2018 (85). For 39/54 (∼72%) small-molecule drugs, there is either direct or indirect evidence that structure-guided lead optimization (typically starting with public domain PDB structures) was employed by one or more biopharmaceutical companies pursuing the drug target. Not surprising, a majority of these 39 cases correspond to 28 small-molecule drugs targeting one or more protein kinases. The bosutinib case study presented above reflects the somewhat serendipitous nature of protein kinase drug discovery. In this case, the target for which drug approval was obtained, Abl, was not Src, the protein for which the compound was originally intended. It is not unusual for protein kinase inhibitors to interdict the activity of more than one closely related protein kinase with beneficial effects in the clinic. Imatinib, for example, was originally approved for treatment of Ph+ CML targeting Abl. Following extraordinarily successful initial clinical outcomes (86), imatinib was shown to inhibit another protein kinase known as Kit, mutants of which cause gastrointestinal stromal tumors (GIST; (87)). Subsequent clinical trials with individuals diagnosed with metastatic GIST allowed the sponsor company Novartis to obtain a US FDA line-extension approval for imatinib in 2012, substantially increasing the number of patients benefiting from the drug with a commensurate impact on sales.

Two additional case studies follow illustrating the impact of academic and industrial structural biologists and PDB data on discovery of three dual cyclin-dependent kinase 4/cyclin-dependent kinase 6 (CDK4/CDK6) inhibitors and an isocitrate dehydrogenase 2 (IDH2) inhibitor.

### CDK4/CDK6 case study

**Figure 3:**
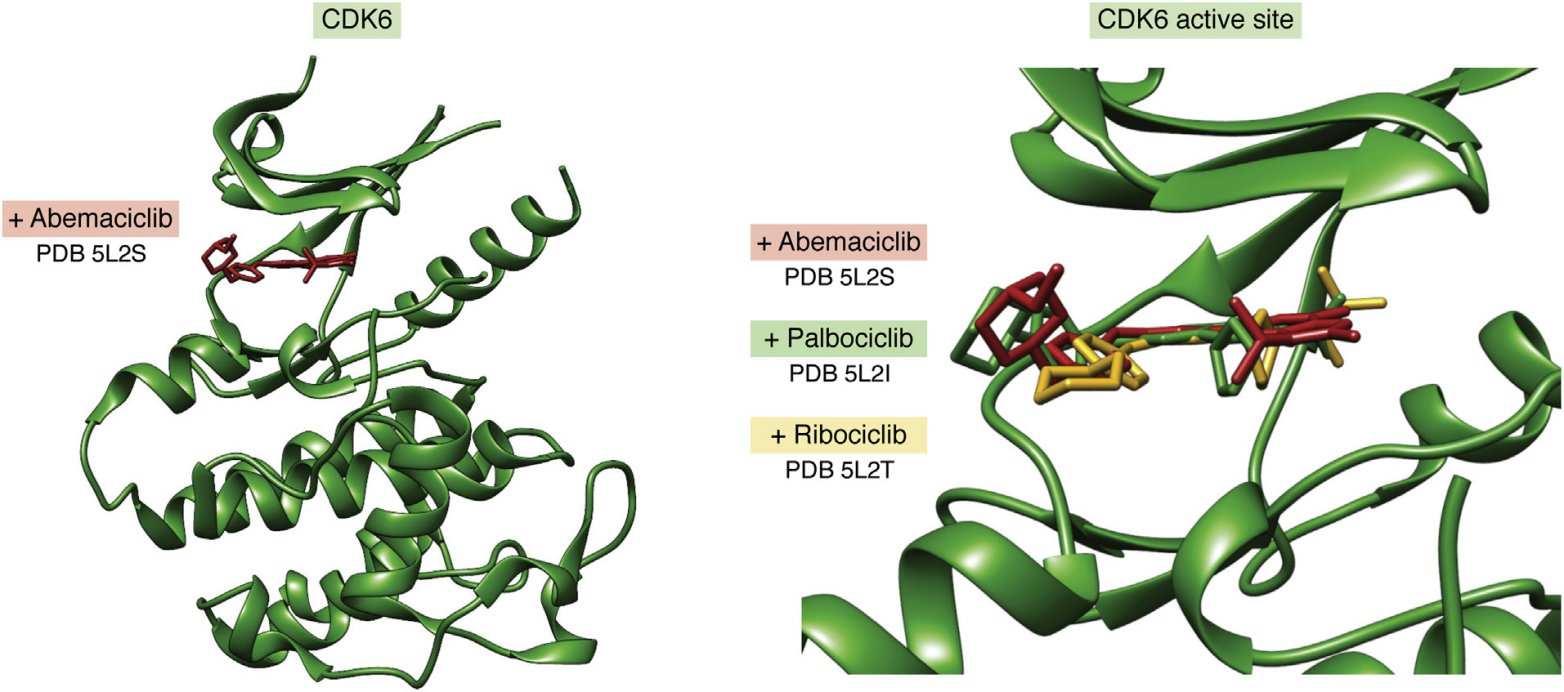
**Hinge-binding inhibitors targeting two cyclin-dependent kinases.***Left*, CDK6 (*green*) bound to abemaciclib (*red*) (PDB ID: 5L2s (91)). *Right*, active site of CDK6 (PDB ID: 5L2s (91)) showing bound abemaciclib (*red*; PDB ID: 5l2s (91)), overlaid with palbociclib (*green*; PDB ID: 5L2i (91)) and ribociclib (*yellow*; PDB ID: 5L2t. (91)).

Among the 28 kinase inhibitors that were identified as products of structure-guided drug discovery are three compounds (palbociclib, ribociclib, and abemaciclib) approved for treatment of breast cancer that target CDK4 and CDK6. These two closely related cyclin-dependent kinases control progression through the G1 phase of the cell cycle, playing central roles in cell proliferation and tumorigenesis. Discovery and near simultaneous approval of the dual inhibitors resulted from parallel efforts by three large biopharmaceutical companies (Pfizer, Novartis, and Eli Lilly and Co). They competed head to head targeting the hinge regions of the two closely related enzymes. Each discovery team would have relied on open access to tens of previously determined CDK structures in the PDB. They would also have been aided by open access to thousands of other protein kinase structures archived in the PDB. Most of these structures were contributed by academic researchers (earliest protein kinase PDB ID released 1993: 2cpk (88); earliest CDK4 PDB ID released 2009: 2w9f (89); earliest CDK6 PDB ID released 1999: 1bi8 (90)). Thanks to Pfizer, cocrystal structures of each dual inhibitor bound to CDK6 are available from the PDB (palbociclib PDB ID: 5L2i (91); ribociclib PDB ID: 5L2t (91); and abemaciclib PDB ID: 5L2s (91)). Close inspection of the modes of inhibitor binding revealed both common (*i.e.*, hydrogen bonding engagement of the hinge region) and disparate features of CDK6-ligand interactions for the three inhibitors (Fig. 3).

This case study also exemplifies how structure-guided lead optimization can be used to discover chemically distinct drug candidates that block the same enzyme active site, allowing each sponsor company to secure its own composition of matter patent protection. For oncology patients, availability of multiple drugs targeting the same protein can be beneficial. A given patient may tolerate side effects (due to off-target binding) of one drug better than those of another. For example, side effect profiles are known to impact patient adherence to treatment of PH+ CML using imatinib and other small-molecule inhibitors of Abl (92).

### IDH2 case study

IDH2 is a homodimeric, NADP(+)-dependent, mitochondrial enzyme responsible for catalyzing oxidative decarboxylation of isocitrate to 2-oxoglutarate in mitochondria. The first PDB structure of a mammalian IDH2 (porcine, 96% identical in amino acid sequence to human) was deposited to the PDB in 2002 by academic researchers (PDB ID: 1lwd (93)). No similar structures were publicly disclosed until 2013, when a small biotechnology company (Agios, Inc) published the structure of the R140Q mutant form of human IDH2 bound to a small-molecule inhibitor (PDB ID: 4ja8 (94)). IDH2 was pursued as a drug discovery target because certain point mutations in IDH2 confer a gain of function on malignant cells, resulting in accumulation and secretion of the oncometabolite (*R*)-2-hydroxyglutarate (reviewed in (95)). In 2018, the PDB archive housed six MX structures of human IDH2, all of which were contributed by biopharmaceutical companies (*i.e.*, Novartis or Agios). A structure-guided drug discovery campaign at Agios yielded enasidenib (PDB ID: 5i96 (96)), which was approved by US FDA in 2017 for treatment of relapsed or refractory acute myeloid leukemia in individuals with specific mutations of the IDH2 gene confirmed by an FDA-approved diagnostic test.

**Figure 4:**
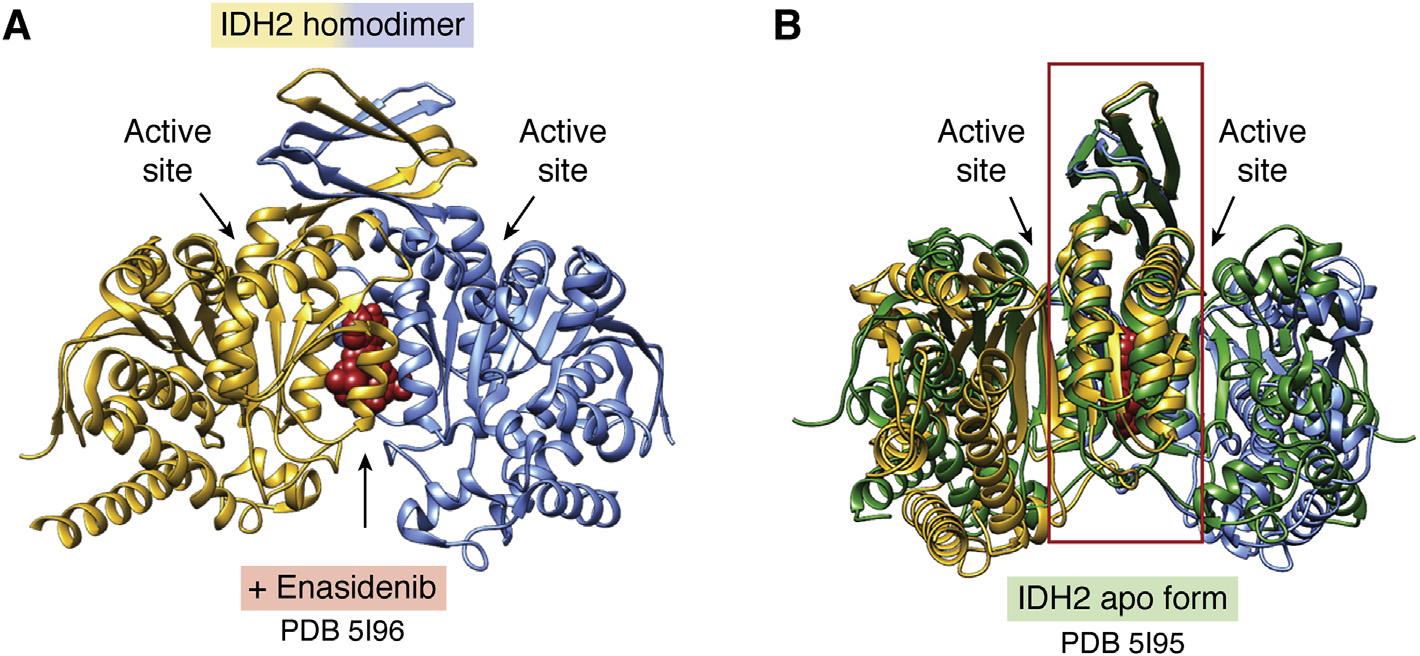
***A*, allosteric inhibitor enasidenib (*red*) targeting homodimeric IDH2 (*yellow* and *blue monomers*; PDB ID: 5i96** (96)**).***B*, superposition of apo (*green* PDB ID: 5i95 (96)) and inhibited (*yellow* and *blue monomers*; PDB ID: 5i96 (96)) forms of IDH1 computed by overlaying the central domains (boxed in *red*). *Arrows* indicate the locations of the two active sites. The view in (*B*) is rotated ∼45° about the vertical relative to (*A*).

Figure 4, *A* and *B* illustrate the allosteric mechanism by which enasidenib inhibits the R140Q mutant form of IDH2. The dimeric enzyme consists of two central domains that participate in dimerization and two pairs of flanking domains. A single enasidenib molecule binds to a site deep within the dimer interface well away from either of enzyme active site (Fig. 4*A*). The inhibited form of the enzyme remains homodimeric, and neither the central nor the two flanking domains undergo significant conformational changes due to drug binding (C*α* root-mean-square deviations for each domain range from 0.8 to 1.0 Å). Instead, a Rube Goldberg Machine-like action at a distance (see https://www.rubegoldberg.com) initiated by drug binding causes the entrances to the two active sites to be splayed open (ASN141-C*α* to HIS175-C*α* separation increases from 5.7 to 9.0 to 10.0 Å), thereby compromising substrate binding.

## Conclusion

Over the next 50 years, we can expect the PDB archive to grow in size and importance as it provides ever larger and more diverse communities of data consumers with open access to well-validated, expertly biocurated 3D structures of biological macromolecules. wwPDB biocurators are already seeing significant flows of new structures coming into the PDB from X-ray free-electron laser studies of enzymes in action (97) and cryo-electron tomography structures of macromolecular machines at work inside living cells that have been caught in the act by flash freezing (98). Both of these rapidly evolving experimental techniques promise to reveal more about the inner workings of the natural world and bring new drug targets into focus. Looking further ahead, integrative (or hybrid) methods (99, 100) will bring 3D structures of even greater import into the PDB over the coming decades. Integrative methods do not rely on a single established method (be it MX, 3DEM, or NMR spectroscopy). Instead, they combine complementary techniques of measurement and computational analysis to resolve very large, complex macromolecular systems, some of which will be the drug targets of tomorrow.

In closing, I would be remiss if I did not highlight the myriad ways in which structural biologists and the wwPDB partners are working together to combat the COVID-19 pandemic. Since late January 2020, more than 1000 structures of SARS-CoV-2 proteins have been deposited into the PDB (http://rcsb.org/covid19). The vast majority of these structures were publicly released well before their appearance in the scientific literature. They are contributing to the fight against the virus in real time (101). More than 100 PDB structures of the viral spike surface glycoprotein are informing successful vaccine design efforts and enabling atomic-level 3D characterization of neutralizing antibodies in approved by the US FDA for passive immunization. Equally important are literally 100s of cocrystal structures of viral proteins bound to small-molecule ligands (102). Strikingly similar among *coronaviridae*, various essential enzymes (main protease, papain-like proteinase, RNA-dependent RNA polymerase, etc.) represent attractive targets for structure-guided discovery of small-molecule antiviral drugs (103, 104). Finally, PDB data are enabling insight into the evolution of SARS-CoV-2 proteins (105) as they change in amino acid sequence and 3D structure, sometimes rendering the virus more transmissible and possibly even deadlier.

## Conflict of interest

The author declares no conflicts of interest with the contents of this article.
